# Dexamethasone for the treatment of traumatic brain injured patients with brain contusions and pericontusional edema: Study protocol for a prospective, randomized and double blind trial

**DOI:** 10.1097/MD.0000000000024206

**Published:** 2021-01-22

**Authors:** Jon Pérez-Bárcena, Ana María Castaño-León, Alfonso Lagares Gómez-Abascal, Jesús Abelardo Barea-Mendoza, Blanca Navarro Maín, Jaume Pomar Pons, Leonor del Mar Periañez Párraga, Javier Ibáñez Domínguez, Mario Chico-Fernández, Juan Antonio Llompart-Pou, Guillem Frontera Juan

**Affiliations:** a Intensive Care Unit, Hospital Universitari Son Espases, Palma de Mallorca; b Department of Neurosurgery, Hospital Universitario 12 de Octubre, Instituto de Investigación Sanitaria Hospital 12 de Octubre (imas12), Universidad Complutense de Madrid; c Intensive Care Unit, Hospital Universitario 12 de Octubre, Madrid; d Neuropsychology and Cognition Research Group, Research Institute on Health IDISBA & IUNICS-UIB, Palma de Mallorca; e Phamacy Department, Hospital Universitari Son Espases; f Department of Neurosurgery, Hospital Universitari Son Espases; g Research Institute on Health IDISBA, Hospital Universitari Son Espases, Institut d’Investigació Sanitària Illes Balears (IdISBa), Palma de Mallorca.

**Keywords:** brain contusions, dexamethasone, pericontusional edema, traumatic brain injury

## Abstract

**Background:**

Traumatic brain injury (TBI) constitutes a leading cause of death and disability. Patients with TBI and cerebral contusions developing pericontusional edema are occasionally given dexamethasone on the belief that this edema is similar to that of tumors, in which the beneficial effect of dexamethasone has been demonstrated.

**Methods:**

The DEXCON TBI trial is a multicenter, pragmatic, randomized, triple-blind, placebo controlled trial to quantify the effects of dexamethasone on the prognosis of TBI patients with brain contusions and pericontusional edema. Adult patients who fulfill the elegibility criteria will be randomized to dexamethasone/placebo in a short and descending course: 4 mg/6 h (2 days); 4 mg/8 hours (2 days); 2 mg/6 hours (2 days); 2 mg/8 hours (2 days); 1 mg/8 hours (2 days); 1 mg/12 hours (2 days). The primary outcome is the Glasgow Scale Outcome Extended (GOSE) performed 1 month and 6 months after TBI. Secondary outcomes are: number of episodes of neurological deterioration; symptoms associated with TBI; adverse events; volume of pericontusional edema before and after 12 days of treatment; results of the neuropsychological tests one month and 6 months after TBI. The main analysis will be on an “intention-to-treat” basis. Logistic regression will estimate the effect of dexamethasone/placebo on GOSE at one month and at 6 months, dichotomized in unfavorable outcome (GOSE 1–6) and favorable outcome (GOSE 7–8). Efficacy will also be analyzed using the 'sliding dichotomy’. An interim and safety analysis will be performed including patients recruited during the first year to calculate the conditional power. A study with 600 patients would have 80% power (2 sided alpha = 5%) to detect a 12% absolute increase (from 50% to 62%) in good recovery.

**Discussion:**

This is a confirmative trial to elucidate the therapeutic efficacy of dexamethasone in a very specific group of TBI patients: patients with brain contusions and pericontusional edema. This trial could become an important milestone for TBI patients as nowadays there is no effective treatment in this type of patients.

**Trial Registration:**

eudraCT: 2019–004038–41; Clinical Trials.gov: NCT04303065.

## Abstract 2

Supplemental Digital Content is available in the text

## 1 Introduction

Traumatic brain injury (TBI) is defined as an alteration in brain function caused by an external force. It is usually classified according to the neurological examination obtained by the Glasgow Coma Score exam (GCS) in mild TBI (GCS 14–15 points), moderate (GCS 9–13 points) or severe (GCS less than 8 points). TBI is a global public health problem as there are more than 50 million cases of TBI per year wordlwide,^[1]^ constituting one of the main causes of death and disability secondary to trauma and causing a great emotional and economic burden for the patients themselves, their families and society. Data published by Eurostat from 24 European countries showed that in 2012 there were 1.5 million hospital discharges and 57,000 deaths attributable to TBI.^[2]^ In 2010, the costs associated with TBI in Europe were estimated at 33 billion euros.^[3]^ In addition, the incidence of TBI has increased in recent years due to the use of motor vehicles in developing countries and the increase in ground-level falls in an increasingly aging population in developed countries.^[1]^

In recent years, the prognosis of this type of patients has not varied substantially.^[4]^ Various explanations for this observation have been described. One reason could be the increase in age in patients with TBI. Older patients are more likely to have complications and therefore these complications could mask an improvement in the management of this type of patients and therefore the prognosis.^[5]^ In addition, head trauma is a complex and heterogeneous pathology, and these are 2 of the reasons why the vast majority of clinical trials have not shown beneficial results of drugs so far,^[1,6]^ except the CRASH-3 trial with tranexamic acid.^[7]^ The most cited reasons for this failure in clinical trials^[1,6,8]^ include the heterogeneity of this pathology; the absence of analytically valid biomarkers or surrogate variables, and the lack of variables or study methods sensitive enough to detect differences between groups.

Among the drugs that have been tested in clinical trials in patients with TBI are corticosteroids. Based on the results of the MRC CRASH study,^[9]^ the current Clinical Practice Guidelines^[10]^ do not recommend the administration of high doses of methylprednisolone to improve the prognosis for patients with TBI. However, due to the experience of dexamethasone in patients with brain tumors, this glucocorticoid is still used in neurosurgical patients with various pathologies and its role is currently being reassessed in patients with TBI and chronic subdural hematomas.^[11,12]^

In daily clinical practice, patients with TBI and cerebral contusions developing an area of pericontusional edema are among the patients who are occasionally given dexamethasone on the belief that this edema is similar to that of tumors. Clinically, the beneficial effect of dexamethasone in patients with tumors and peritumoral edema, which is primarily vasogenic, has been well demonstrated.^[13]^

Cerebral edema is classified as cytotoxic and vasogenic. Cytotoxic edema is primarily intracellular and appears after ischemic insults. In vasogenic edema, however, water has an extracellular location and appears when there is a disruption or an abnormal increase in the permeability of the blood brain barrier.^[14]^ Published studies have described the presence of a mixed component (vasogenic and cytotoxic) in patients with TBI and cerebral contusions.^[15,16]^ Experimental studies have shown that the alteration of the blood brain barrier is biphasic. There is a rapid initial opening phase after the trauma that occurs in the first hours. Subsequently, a second opening phase of the blood brain barrier occurs, which usually begins between 3 to 7 days after the trauma.^[17]^ At the clinical level it has been proven that can take days or weeks for the blood brain barrier to return back to normal.^[17,18]^ For this reason it is possible that there is a therapeutic window for corticosteroids in this type of patients. However, it is well known that corticosteroids have a beneficial effect on vasogenic edema (as in patients with tumors) but none on cytotoxic edema.

Using diffusion tensor magnetic resonance imaging (DT-MRI), our group has shown that the radiological characteristics of the vasogenic edema in patients with brain tumors and cerebral contusions are similar. Specifically, the apparent diffusion coefficient (ADC) and fractional anisotropy (FA) were similar in both groups of patients.^[19]^ We have also observed, using DT-MRI, in a group of 30 patients with TBI, cerebral contusions and pericontusional edema, that the use of dexamethasone at low doses was associated with a reduction in the volume of cerebral edema and improvement in radiological parameters (ADC and FA).^[20]^

For this reason, and taking into account the preliminary results mentioned in the previous paragraph, it is intended to conduct a clinical trial to assess the effect of dexamethasone on the prognosis of TBI patients with brain contusions and pericontusional edema. The idea of guiding the treatment of patients with TBI and cerebral contusions depending on the type of edema can be very useful and a novel way of designing this clinical trial.

To conclude and as described in the third paragraph, the current Clinical Practice Guidelines^[10]^ do not recommend the administration of high doses of methylprednisolone to improve the prognosis for patients with TBI. In our opinion this does not mean that the effect of low doses of dexamethasone (maximum dose of 16 mgr per day) should not be studied in a short and descending course to a selected group of patients such as patients with cerebral contusions and pericontusional edema. This dose is the usual one in neurooncology and there is an extensive experience in its use. In addition, 2 clinical trials with dexamethasone at these doses are already being conducted in patients with TBI but with chronic subdural hematomas.^[11,12]^

## 2 Objective

To estimate the efficacy of dexamethasone, compared to placebo, in patients with TBI and brain contusions.

## 3 Methods

### 3.1 Design of the study

**Figure 1:**
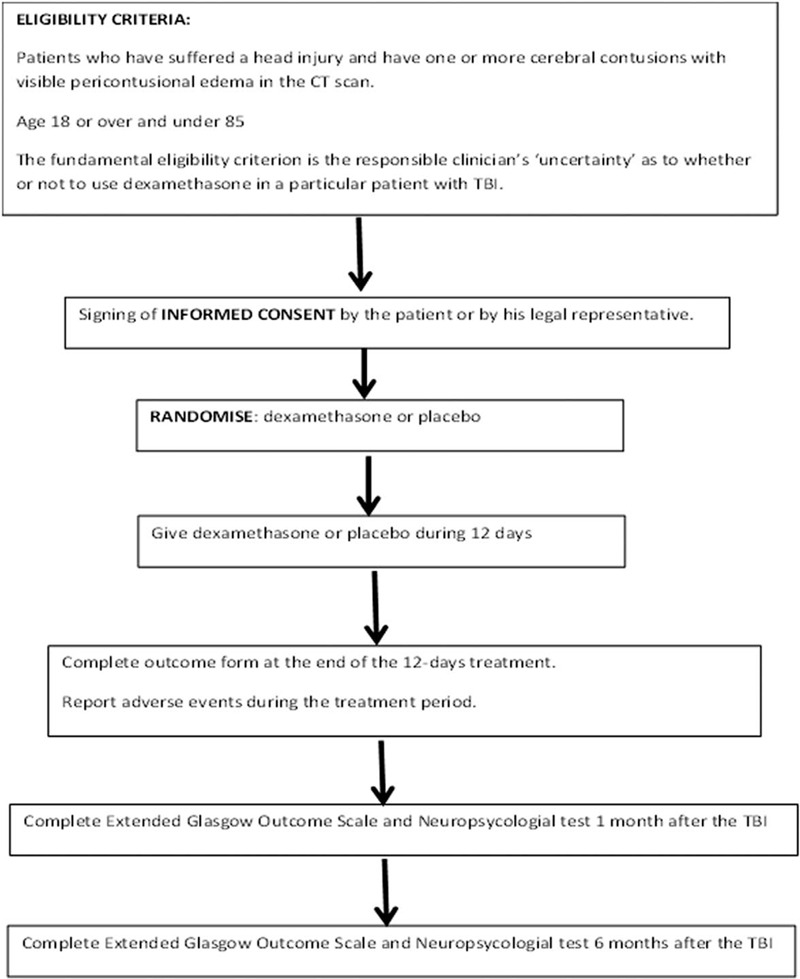
Trial overview.

The DEXCON TBI trial is a multicenter, pragmatic, randomized, triple-blind, placebo controlled trial to quantify the effects of the administration of dexamethasone on the outcome of TBI patients with brain contusions and pericontusional edema. A trial overview is shown in Figure 1.

### 3.2 Pragmatic design and the uncertainty principle

The design will allow us to find out how effective the treatment actually is in routine everyday practice. Ethically, this randomized controlled trial can only be undertaken if there is collective scientific uncertainty about which of the interventions being compared is more likely to benefit patients. However, for an individual clinician to be able to recommend enrolment of a patient into a trial, they must be substantially uncertain about the appropriateness of the trial treatment in that particular patient. The eligibility criteria for the DEXCON trial are based on this uncertainty principle. A patient can be enrolled only if the responsable clinician is substantially uncertain as to which of the trial treatments would be most appropriate for that particular patient. Using the uncertainty principle should allow the process of this trial to be closer to what is customary in normal medical practice.

### 3.3 Ethical considerations, information giving and written informed consent

Ethics approval of the coordinating centre was obtained from Comité de Ética de la Investigación de las Islas Baleares (CI-IB:10/19, eudraCT: 2019–004038–41) and from Comité de Ética de la investigación from the Hospital Universitario 12 de Octubre. The trial has also been approved by the Agencia Española del Medicamento y Productos Sanitarios (AEMPS), which is a public agency depending on the Health Ministry of the Spanish Government.

The GCS score is a method of assessing the level of consciousness in TBI patients. Patients with a GCS score of 15 are generally considered fully conscious, but those with a GCS score of 14 or less may not be fully conscious and would not be mentally capable of giving informed consent to participation in a clinical trial. Besides, a brain contusion is a clinical sign indicating significant brain injury, and patients with this diagnosis would not be physically or mentally capable of giving informed consent to participation in a clinical trial.

Therefore, given that patients are eligible for inclusión in the DEXCON TBI trial if they have TBI with a brain contusion on a CT scan, in most cases they will, by default, be physically or mentally incapable of giving consent.

### 3.4 Prior information giving to the patients relatives

If relatives are present, they will be provided with brief information about the trial. Specifically, the responsible doctor will explain to the relatives that the patient will receive the usual treatments for TBI but that, in addition to these, the patient has been enrolled in a research study that aims to improve the treatment of patients with this condition.

It will be explained that the study is being done to see whether using a drug called dexamethasone will help patients with head injury by reducing the amount of edema and inflammation into the brain, therefore preventing further brain damage. The relative will be informed that the patient will be given a treatment over 12 days of either dexamethasone or a dummy medicine (a capsule which does not contain dexamethasone). The doctor will explain that dexamethasone has been shown to improve outcome in patients with other types of brain injuries, such as tumors, and that, whilst we hope that it will also improve recovery after head injury, at present we cannot be sure about this. If requested, a brief information sheet will be provided. If relatives object to the inclusion of the patient in the trial, their views will be respected. If no relatives are present, the patient can not be included in the study.

### 3.5 Eligible patients

Adults with TBI who fulfill the following inclusión criteria are eligible:

- Patients who have suffered TBI and have one or more cerebral contusions with visible pericontusional edema in the computed tomography (CT) scan.
- Patients with brain contusions in whom non-sugical treatment has been selected initially.
- Age 18 or over and under 85.
- Signing of informed consent by the patient or by his legal representative.

The fundamental eligibility criterion is the responsible clinicians “uncertainty” as to whether or not to use dexamethasone in a particular patient with TBI. This pragmatic approach will allow us to see whether the intervention improves patient outcomes under real-life conditions.

The exclusion criteria are the following:

- Patients with TBI and brain contusions who have required surgery to evacuate the cerebral contusion before randomization.
- Patients with TBI who have required a craniotomy before randomization for any other reason: evacuation of subdural, epidural hematoma or depressed skull fracture.
- Patients with an extracranial Injury Severity Score greater than 18 points.
- Patients in whom the use of corticosteroids is contraindicated.
- Patients who take oral corticosteroids chronically.
- Patients included in another clinical trial.
- Known intolerance or hypersensitivity to dexamethasone.
- Patients with allergy or intolerance to the following excipients contained in dexamethasone/placebo capsules: lactose, corn starch or microcrystalline cellulose.
- Patients with a history of psychotic disorders.
- Patients with inability to take medication orally due to swallowing problems in which it is not indicated to place a nasogastric tube.
- Pregnant or breastfeeding patients.
- Patients in a GCS 3 points situation with bilateral fixed dilated pupils.
- Patients with associated spinal cord injuries.
- Patient with any systemic condition that contraindicates the use of corticosteroids.

### 3.6 Randomization

To ensure a balanced sample size across groups over time, the statistician of the study will apply the block randomization method with a block size of 2 (2 possible sequences: AB and BA will be generated with the statistical package R, version 3.5.3, a list for each hospital). A limited number of treatments will be available in each hospital, sent from the Pharmacy Department of Hospital Universitari Son Espases, which will be numbered and will be assigned in a correlative manner.

Patients, clinical staff, evaluators of the results as well as the statistical team will be blinded to the assignment of medications.

Only the pharmacist, who masks the capsules and labels the containers, will know the assignement of the codes, and will keep them blind until the last phase of the study, after the main statistical analysis, or when unmasking is required.

Once it is verified that the patient meets all the inclusion criteria and none of the exclusion criteria, and after signing the informed consent by the patient himself or by the closest relative, the doctor responsible for the patient will correlatively assign the number to the patient.

### 3.7 Settings

Initially, the study will be carried out in Hospital Universitari Son Espases (Palma de Mallorca) and Hospital Universitario 12 de Octubre (Madrid). During the first year, all possible patients will be tried to be recruited. A minimum of 60 patients are expected between the 2 hospitals.

If after the first year interim analysis the Trial Steering Committee (TSC) decides to continue the trial, recruitment will begin in the hospitals included in the Spanish Trauma ICU Registry (RETRAUCI Network) which includes 56 public hospitals.

### 3.8 Outcome measures

**Figure 2:**
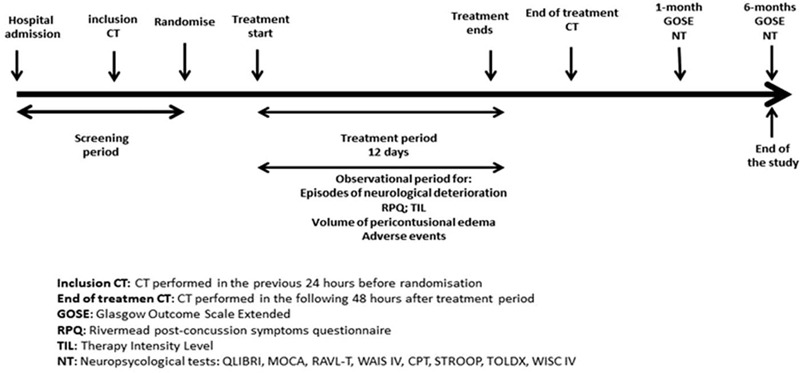
Measures of outcome during the trial.

After a patient has been randomized, the outcome will be collected even if the trial treatment is interrupted or is not actually given (Fig. 2)

#### 3.8.1 Primary outcome

The primary outcome is the functional status (outcome) of TBI patients one and 6 months after the trauma according to the Glasgow Scale Outcome Extended (GOSE).

#### 3.8.2 Secondary outcomes

- Number of episodes of neurological deterioration in both groups of patients during the 12 days of treatment (Supplemental data 1).
- Symptoms associated with TBI in both groups of patients during the 12 days of treatment.
- Volume of pericontusional edema before and after 12 days of treatment in both groups of patients.
- Presence of adverse events between the 2 groups during the 12 days of treatment.
- Neuropsychological tests between the 2 groups of patients one month and 6 months after the TBI.

### 3.9 Estimated event rate

In our previous studies we have observed that among TBI patients with brain contusions not treated with dexamethasone, the proportion of patients with good recovery (GOSE 7 and 8) was 50%.

### 3.10 Sample size and treatment effect size that should be detectable

Since the data available in this type of patients with this treatment is scarce and inconclusive, we will perform a randomized, triple-blind, placebo controlled trial with an interim futility analysis (interim analysis). The primary endpoint for the DEXCON TBI trial is the good recovery (GOSE 7–8) one month after the TBI.

A study with 600 patients would have about 80% power (2 sided alpha = 5%) to detect a 12% absolute increased (from 50% to 62%) in good recovery (GOSE 7–8). It includes an inflation of 10% due to non-adherence (withdrawal of consent, loss to follow-up, cross-over or change of treatment).

### 3.11 Treatment

Dexamethasone will be compared with matching placebo.

### 3.12 Dose selection

Dexamethasone doses are those commonly used in neurooncology and there is an extensive experience in its use. It will be a short and descending course: 4 mg/6 hours (2 days); 4 mg/8 hours (2 days); 2 mg/6 hours (2 days); 2 mg/8 hours (2 days); 1 mg/8 hours (2 days); 1 mg/12 hours (2 days).

### 3.13 Drug manufacture, blinding and supply of trial treatment

Dexamethasone (Fortecortin) will be acquired from ERN, SA. Laboratories (Barcelona, Spain). The Pharmacy Department of the Hospital Universitari Son Espases will be in charge of developing and conditioning the 4 mg, 2 mg and 1 mg dexamethasone/placebo capsules needed for 12 days of treatment, keeping the researchers blind.

The preparation and conditioning of the capsules will be carried out following the standardized work procedures of the pharmaceutical laboratory and its quality controls, previously authorized by the AEMPS.

The Pharmacy Department of the Hospital Universitari Son Espases will be responsible for identifying the containers and sending them by courier to the participating hospitals. A record of the dispensing of test samples will be kept and will be sent in acknowledgment of receipt for control.

Patients, clinical staff, evaluators of the results as well as the statistical team that will perform the analysis will not know the assigned treatment. A randomization list will be created for each hospital. The randomization sequence will only be known by the Pharmacy Department of the Hospital Universitari Son Espases.

### 3.14 Administration of trial treatment

Each patient will receive 3 containers with the correspondingly identified doses, and an information sheet explaining dose and frequency for proper oral administration.

### 3.15 Other treatments for traumatic brain injury

There is a wide spectrum of treatments for TBI. As the trial will be conducted in Spain, each participating site should follow its own clinical guidelines for the treatment of TBI patients. There is no need to withhold any clinically indicated treatment in this trial. Dexamethasone or placebo would be provided as an additional treatment to the usual management of TBI.

### 3.16 Unblinding

In general there should be no need to unblind the allocated treatment. Unblinding should be done only in those rare cases when the clinician believes that clinical management depends importantly upon knowledge of whether the patient received dexamethasone or placebo. In those few cases when urgent unblinding is considered necessary, a 24-h telephone service will be available. The caller will be told whether the patient received dexamethasone or placebo.

### 3.17 Data collection and management

This trial will be coordinated from the Hospital Universitari Son Espases. Data will be collected at each site by local investigators and sent to the TSC. These data will be collected from the patients routine medical records and no special tests will be required.

All data on adverse events, including those routinely collected as outcomes, will be collected and reported as recquired by the relevant authorities.

Data will be collected by the investigator on the paper Case Report Forms (CRFs) and transmitted to the TSC through the Research Electronic Data Capture (REDCap). REDCap is a secure web application for building and managing online surveys and databases. Original paper CRFs will remain at each trial site. The data will be used in accordance with local law and ethics committee approval.

### 3.18 End of trial for participants

For the recruited patients the trial ends at death or at 6-month follow-up, whichever occurs first. If during the treatment phase a patient develops an adverse event, the trial drug should be stopped, the patient treated in line with local procedures, and then followed up.

The trial may be terminated early by the TSC. The independent Data Monitoring Committee (DMC) may give advice/recommendation for the early termination of the trial but the TSC is responsible for the final decision.

### 3.19 Adverse events

Please see supplemental data 2.

### 3.20 Analysis

The primary analysis will compare all patients allocated to dexamethasone versus those allocated to placebo, on an “intention-to-treat” basis. A descriptive analysis of the baseline variables will be made for each treatment group, followed by a comparison between both groups.

In the secondary analysis a logistic regression model will be built to estimate the effect of dexamethasone and placebo on the patient's functional status measured with the GOSE at one month and at 6 months. The scale will be dichotomized in unfavorable outcome (GOSE 1–6) and favorable outcome (GOSE 7–8). In this analysis the primary outcome will be adjusted by age, pupil reactivity, blood pressure and GCS. To choose a model, the adequacy and goodness of fit will be measured.

Since the severity of the initial injury will significantly determine the final outcome of the patient, regardless of any treatment, the results of this study will be analyzed using the “sliding dichotomy”. According to this analysis, patients with a less severe initial injury should have a better recovery than those with a severe initial injury. For example, a moderate disability in a patient for whom no more than death or severe disability could be expected is considered a good outcome, and vice versa, consider a moderate disability in a patient with excellent initial prognosis as poor outcome. Patients with a severe initial injury (GCS score of 4 to 5 or, or with a GCS motor score of 2 to 3) will be considered to have a favorable outcome if the 6-month GOS-E score is 3 or higher. Patients with a moderate-to-severe initial injury (GCS score of 6 to 8 or, GCS motor score of 4 to 5) will be considered to have a favorable outcome if the 6-month GOS-E score is 5 or higher, and those with a less initial injury (GCS score of 9 to 12, GCS motor score of 6) will be considered to have a favorable outcome if the 6-month GOS-E score is 7 or higher.

The purpose of the sliding dichotomy approach is to increase the sensitivity (to lower the false negative rate) of the analysis. All these variables will also be analyzed in a pre-specified subgroup analysis comparing patients with a pericontusional edema volume of more than 10 ml in the pre-inclusion CT scan in the study with those who have less than 10 ml of edema volume in the same CT study.

### 3.21 Interim analysis

Since the data available in this type of patients receiving this treatment are scarce and inconclusive as to the primary outcome variable, we will perform an interim analysis. A statistician (GF) will blindly perform an interim analysis calculating the conditional power, an approach that quantifies the probability of obtaining a significant result at the end of the study given the data available after one year enrolling patients.

Since the probablilty chosen for futility in this trial is very small (<0.15), it can be concluded that it would be useless to continue the trial. This interim analysis will be performed at the end of the first year since the inception of the trial, when it is estimated that about 100 patients will have been recruited. At the same time, a safety analysis will be also performed, focused on the adverse effects collected during the first year.

The results will be assessed and discussed by an independent DMC, formed by one statistician and 2 experts in TBI, who will inform and make recommendations to the TSC, which will finally make the decisions.

### 3.22 Independent Data Monitoring Committee (DMC) and Trial steering committee (TSC)

Please see supplemental data 3.

### 3.23 Follow-up

No extra tests are required for the trial but a case report form should be completed at the end of the 12- day treatment, or at death or hospital discharge if either happens sooner. The GOSE and the neuropsychological evaluations will be performed one month and 6 months after the TBI. Both evaluations will be performed by clinical neuropsychologists, and the protocol used will always follow the same process. The neuropsychological tests that will be performed are described in the Supplemental data.

## 4 Discussion

The current trial is a confirmative trial to elucidate the therapeutic efficacy of dexamethasone in a very specific group of TBI patients: patients with brain contusions and pericontusional edema. This trial could become an important milestone for TBI patients as nowadays there is no effective treatment in this type of patients. Patient recruitment is planned to take place during the first year in Hospital Universitari Son Espases (Palma de Mallorca, Spain) and Hospital Universitario 12 de Octubre (Madrid, Spain), and is active starting August 2020.

## Author contributions

**Conceptualization:** Jon Pérez-Bárcena, Ana María Castaño-León, Alfonso Lagares Gómez-Abascal, Javier Ibáñez Domínguez, Guillem Frontera Juan.

**Data curation:** Jon Pérez-Bárcena, Ana María Castaño-León, Jesús Abelardo Barea-Mendoza, Mario Chico-Fernández, Juan Antonio Llompart-Pou.

**Formal analysis:** Guillem Frontera Juan.

**Funding acquisition:** Jon Pérez-Bárcena.

**Investigation:** Jon Pérez-Bárcena, Ana María Castaño-León, Alfonso Lagares Gómez-Abascal, Jesús Abelardo Barea-Mendoza, Blanca Navarro Maín, Jaume Pomar Pons, Javier Ibáñez Domínguez, Mario Chico-Fernández, Juan Antonio Llompart-Pou.

**Methodology:** Jon Pérez-Bárcena, Ana María Castaño-León, Alfonso Lagares Gómez-Abascal, Jesús Abelardo Barea-Mendoza, Blanca Navarro Maín, Jaume Pomar Pons, Leonor del Mar Periáñez, Javier Ibáñez Domínguez, Guillem Frontera Juan.

**Project administration:** Jon Pérez-Bárcena, Alfonso Lagares Gómez-Abascal, Javier Ibáñez Domínguez, Guillem Frontera Juan.

**Resources:** Ana María Castaño-León, Leonor del Mar Periáñez.

**Supervision:** Mario Chico-Fernández, Juan Antonio Llompart-Pou.

**Writing – original draft:** Jon Pérez-Bárcena.

**Writing – review & editing:** Jon Pérez-Bárcena, Ana María Castaño-León, Alfonso Lagares Gómez-Abascal, Jesús Abelardo Barea-Mendoza, Blanca Navarro Maín, Jaume Pomar Pons, Leonor del Mar Periáñez, Javier Ibáñez Domínguez, Mario Chico-Fernández, Juan Antonio Llompart-Pou, Guillem Frontera Juan.

## Supplementary Material

Supplemental Digital Content

## Supplementary Material

Supplemental Digital Content

## Supplementary Material

Supplemental Digital Content
