# Supplementary material for: Dexamethasone for the treatment of traumatic brain injured patients with brain contusions and pericontusional edema: Study protocol for a prospective, randomized and double blind trial
Source: Medicine (Baltimore). 2021 Jan 22;100(3):e24206. doi: 10.1097/MD.0000000000024206 (PMC7837989; doi:10.1097/MD.0000000000024206)
Supplement: Supplemental Digital Content [file medi-100-e24206-s001.docx]

**SUPPLEMENTAL DATA 1.**

**Primary Outcome measurement**

The primary outcome is the Glasgow Outcome Scale Extended (GOSE) performed one month and 6 months after trauma. This scale will be dichotomized in favorable outcome (GOSE 7-8 points) and unfavorable outcome (GOSE≤6 points)

**Secondary Outcome measurements**

*-Compare the number of episodes of neurological deterioration in both groups of patients during the 12 days of treatment.*

The number of episodes of neurological impairment will be detected by the Glasgow Coma Scale and the abbreviated NIHSS scale, during the 12 days of treatment.

An episode of neurological impairment is defined as a worsening of at least 2 points on the Glasgow Coma Scale or the abbreviated NIHSS scale, which lasts at least 2 hours, and which cannot be fully attributed to other causes other than cerebral edema. In order to rule out other causes of neurological deterioration, complementary tests will be performed according to the protocol of action of each hospital. Among these tests are an arterial blood gas analysis, blood test with blood count and biochemistry, electroencephalogram and a cranial CT scan.

Baseline scores to compare subsequent neurological examinations will be performed 30 minutes before starting the study medication. Subsequently, the reference scores will depend on the patient's clinical evolution. After sustained episodes of improvement or worsening in the patient's clinical situation, the baseline scores will be recalculated to reflect the best score of the Glasgow Coma Scale and the NIHSS before a possible episode of neurological impairment. In those patients in whom it is not possible to perform neurological examinations, the reason will be collected in the Investigator’s Study File.

The neurological exams will be performed 3 times per day during the 12 days of treatment.

*-Compare the symptoms associated with TBI in both groups of patients during the 12 days of treatment.*

The symptoms associated with TBI will be studied daily through the Rivermead Post Concussion Symptom (RPQ) scale. The RPQ is a questionnaire that measures the severity of symptoms associated with TBI. The questionnaire contains 16 symptoms and the patient is asked to quantify these symptoms during the past 24 hours.

In each case, the symptoms are compared with the severity of these symptoms before the trauma. The symptoms range from 0 to 4 points: 0: it has not presented such a symptom; 1: it has not been a bigger problem than before the TBI; 2: slight problem; 3: moderate problem; 4: serious problem.

The RPQ scale will be performed once daily during the 12 days of treatment.

*-Compare the volume of pericontusional edema before and after 12 days of treatment in both groups of patients.*

In each center, researchers will record the volume of the lesions including the hemorrhagic portion and the pericontusional edema using the ABC / 2 methodology. In the case of multiple contusions, each of them will be measured separately up to a number of 3, starting with the largest.

The image will be analyzed using a standard window for all cases (window level 60 and width of 100 UH), thus ensuring a similar gray scale in the different centers. A measurement of the largest diameter (A) will be made in each contusion, followed by the largest perpendicular diameter to A (B) and the number of cuts in which the contusion (hyperintense area and perilesional hypodense area) can be identified (C). The total volume of the bruises will be equal to the sum of the individual volumes of each one, whose volume results from multiplying the three diameters and dividing them by two. Once this process has been carried out, a measurement will only be made of the hyperdense portion of the contusions to establish the volume of the edema:

Edema volume = total volume-volume hyperdensity.

This procedure will be performed again centrally in the Hospital Universitario 12 de Octubre, Madrid. Previously, the patient's data will be anonymized and the images will be sent through a web platform with a secure server for review. In a central way, the total volume of the contusion will be established as well as the volume of the edema by means of a semi-automatic method using the Analyze software (version 10.0; Analyze Direct, Stilwell, Kansas, USA) of which a license is available. The method consists of a semi-automatic analysis in which in each axial section a central voxel is selected and thanks to the density selection, voxels with similar density characteristics are selected.

The performance of measurements in each hospital and subsequent centralization of the images will allow to establish the inter-observer and inter-method reproducibility of the measurement of the edema associated with the contusions, in addition to estimating in two ways the difference of measurements at two time points throughout the study and after an intervention.

*-Compare the presence of adverse events between the two groups during the 12 days of treatment.*

Those adverse effects that have special interest and serious adverse effects will be collected. Side effects of special interest are:

-Metabolic: Hyperglycemia will be especially monitored. The maximum value of capillary glycemia will be recorded daily. The amount of daily insulin that the patient needs to control de glucose level will also be collected.

-Psychiatric: presence of psychotic symptoms using the Confusion Assessment Method (CAM).

-Digestive: Episodes of gastrointestinal hemorrhage; Episodes of epigastric pain, vomiting or reflux symptoms.

-Infectious: Presence of new infectious episodes. Infectious episodes will be confirmed in accordance with the Centers for Disease Control (CDC) criteria and mainly based on microbiological criteria.

*-Compare the results of the neuropsychological tests between the two groups of patients one month and 6 months after the TBI.*

Two neuropsychological evaluations will be performed: 1 month and 6 months after the TBI. Both evaluations will be performed by clinical neuropsychologists, and the protocol to be used will always follow the same procedure and tests. The tests that integrate this protocol are described below:

-The MOCA (Montreal Cognitive Assessment): originally designed as a rapid screening instrument for mild cognitive dysfunction. It assesses different cognitive domains: attention and concentration, executive functions, memory, language, visuoconstructional skills, conceptual thinking, calculations and orientation.

- Rey Auditory Verbal Learning Test (RAVLT) is one of the most widely used word learning tests in clinical research and practice. Five presentations of a 15-word list are given, each followed by attempted recall. This is followed by a second 15-word list (list B), followed by recall of list A, and delayed recall and recognition are also tested.

- Coding (WAIS-IV Battery subtest): essentially aims to assess processing speed, associative memory, graphomotor speed.

- Computerized Continuous Continued Test (CPT) The CSAT-II RESEARCH VERSION: this version is used to evaluate sustained attention, discrimination, types of errors made, motor response style and response speed during the task.

- Stroop test: This is a psychological test linked especially to neuropsychology that measures the level of interference generated by automatisms in the performance of a task.

- Digit Span (WAIS-IV Battery subtest): essentially measures auditory working memory and your ability to record, maintain and manipulate auditory information consciously.

- Test Tower of London-Drexel University version test (TOLDX): measures executive planning ability in subjects with frontal lobe injury. Traditionally used as a planning and problem-solving measure.

- Quality of Life after brain injury (QOLIBRI) Spanish version: to measure quality of life after TBI. Through these scales and questionnaires, what is intended is to obtain information obtained, preferably, from the patient himself and simultaneously from a family member or friend (proxy) in order to corroborate the information provided by the patient himself.
