# Supplementary material for: Dexamethasone for the treatment of traumatic brain injured patients with brain contusions and pericontusional edema: Study protocol for a prospective, randomized and double blind trial
Source: Medicine (Baltimore). 2021 Jan 22;100(3):e24206. doi: 10.1097/MD.0000000000024206 (PMC7837989; doi:10.1097/MD.0000000000024206)
Supplement: Supplemental Digital Content [file medi-100-e24206-s002.docx]

**SUPPLEMENTAL DATA 2.**

**Safety and Adverse events**

Dexamethasone has a well-documented safety profile. Nevertheless, data on adverse events, such as infectious complications, hyperglycemia, gastrointestinal bleeding or appearance of psychotic symptoms will be collected as secondary outcomes and will be presented to the independent Data Monitoring Committee (DMC) for blinded review.

**Definitions**

**Adverse events (AE):** Any undesirable and unintended medical occurrence affecting a trial participant during the course of a clinical trial.

**Serious adverse events (SAE):** A serious adverse event (experience) is any undesirable medical occurrence that results in death or is life-threatening or requires inpatient hospitalization or prolongation of existing hospitalization, or results in persistent or significant disability/incapacity.

**Reporting of adverse events for this trial**

Death, life-threatening complications, and prolonged hospital stay which are attributable to the adverse event are pre-specified outcomes to be reported in this trial. This clinical trial is being conducted in an emergency condition using a drug in common use. It is important to consider the natural history of the critical medical event affecting each patient enrolled, the expected complications of this event, and the relevance of the complications to dexamethasone.

Adverse events to be reported using an adverse event reporting form will be limited to those trial outcomes that might occur and be reasonably attributable to the trial drug. Events that are part of the natural history of the primary event of TBI or expected complications of TBI should not be reported as adverse events.

If an SAE occurs, a written report must be submitted within 24 h. The Trial Steering Committee (TSC) will coordinate the reporting of all SAEs to all relevant Regulatory Agencies, Ethics Committees and local investigators as per local legal requirements.

**Monitoring**

The intervention with dexamethasone has marketing authorization in Spain and has been in clinical use for decades. Its safety profile is well established and no significant serious adverse events associated with its use have been identified. The trial will routinely collect data on adverse events which may theoretically be associated with this product and the condition under investigation, and these will be reviewed by the independent DMC.

The trial procedures are based on routine clinical procedures and include (1) the oral administration of the trial drug using routine clinical use; (2) collecting routine clinical information from the medical records; and (3) informed consent. There are no complex procedures or interventions for the participants or investigators in this trial. Clinical management for underlying conditions will remain as per each hospital’s standard protocol.

Investigators/institutions are required to provide direct access to source data/documents for trial related monitoring, audits, ethics committee review and regulatory inspection. All trial-related and source documents must be kept for at least 5 years after the end of the trial.
