# Supplementary material for: Dexamethasone for the treatment of traumatic brain injured patients with brain contusions and pericontusional edema: Study protocol for a prospective, randomized and double blind trial
Source: Medicine (Baltimore). 2021 Jan 22;100(3):e24206. doi: 10.1097/MD.0000000000024206 (PMC7837989; doi:10.1097/MD.0000000000024206)
Supplement: Supplemental Digital Content [file medi-100-e24206-s003.docx]

**SUPPLEMENTAL DATA 3.**

**Recruitment of collaborating investigators**

The trial will recruit hospitals from Spain. Suitable collaborating hospitals and investigators will be assessed in terms of the trauma service that they provide and their ability to conduct the trial.

Before the trial can begin at any site, the local Principal Investigator must agree to adhere to Good Clinical Practice Guidelines and all relevant national regulations. In addition, all relevant regulatory and ethics approvals should be in place before the trial starts at a site.

**Independent Data Monitoring Committee (DMC)**

Dr Pedro Delgado MD. Servicio de Neurocirugía. Hospital Universitario de Burgos. Spain.

Dr Marcelino Sánchez Casado MD, PhD. Servicio de Medicina Intensiva. Hospital Virgen de la Salud. Toledo. Spain

Dr Carlos Campillo Artero MD; PhD. Servei de Salut de les Illes Balears, Palma de Mallorca, Spain, CRES/BSM Universitat Pompeu Fabra, Barcelona Spain.

To provide protection for study participants, an independent DMC has been appointed for this trial to oversee the safety monitoring. The DMC will review, after the first year, accumulating data from the ongoing trial and advise the TSC regarding the continuing safety of current participants and those yet to be recruited, as well as reviewing the validity and scientific merit of the trial. The statistician (GF) will provide (in a blind manner) the analysis service required by the DMC.

**Standard operating procedures**

The DMC has the responsibility for making recommendations to the TSC, while randomization is in progress, to unblind the results. The DMC they will do this if, and only if, the following two conditions are satisfied: (1) the results provide proof beyond reasonable doubt that treatment is on balance either definitely harmful or definitely favourable for all, or for a particular category of participants in terms of the major outcome; and (2) the results, if revealed, would be expected to substantially change the prescribing patterns of clinicians who are already familiar with any other trial results that exist. Exact criteria for “proof beyond reasonable doubt” are not specified by a purely mathematical stopping rule, but they are strongly influenced by such rules.

**Trial steering committee (TSC)**

Dr Jon Pérez Bárcena. Intensive Care Unit. Hospital Universitari Son Espases. Palma de Mallorca.

Dra Ana María Castaño León. Department of Neurosurgery. Hospital Universitario 12 de Octubre Hospital. Madrid.

Dr Alfonso Lagares Gómez-Abascal. Department of Neurosurgery. Hospital Universitario 12 de Octubre. Madrid.

Dr Jesús Abelardo Barea-Mendoza. Intensive Care Unit. Hospital Universitario 12 de Octubre. Madrid.

Dr Javier Ibánez Domínguez. Department of Neurosurgery. Hospital Universitari Son Espases.

Dr Guillem Frontera. Research Unit. Hospital Universitari Son Espases Hospital. Palma de Mallorca.

The role of the TSC is to provide overall supervision of the trial. In particular, the TSC will concentrate on the progress of the trial, adherence to the protocol, patient safety and consideration of new information. The TSC will take responsibility for: a) major decisions such as a need to change the protocol for any reason; b) monitoring and supervising the progress of the trial; c) reviewing relevant information from other sources; d) considering recommendations from the DMC.

When outcome data are available for the participants included during the first year, the TSC will review the rate of recruitment into the trial and the overall event rates. The TSC will consider the extent to which the rate of recruitment and the event rates correspond to those anticipated before the trial and will take whatever action is needed in light of this information.

**Collaborators´responsibilities**

If after the first year interim analysis the TSC recommends to continue the trial, recruitment will begin in the hospitals included in the Spanish Trauma ICU Registry (RETRAUCI Network). This network involves 56 public hospitals that are already actively collaborating. The coordinating center for the DEXCON TBI trial will be Hospital Universitari Son Espases (Palma de Mallorca, Spain).

Coordination within each participating hospital will be through a local Principal Investigator whose responsibility will be detailed in an agreement in advance of starting the trial and will include: ensure all necessary approvals are in place prior to starting the trial; delegate trial-related responsibilities only to suitably trained and qualified personnel; train relevant medical and nursing staff who see TBI patients and ensure that they remain aware of the state of the current knowledge, the trial and its procedures; agree to comply with the final trial protocol and any relevant amendments; ensure that all patients with TBI are considered promptly for the trial; ensure consent is obtained in line with local approved procedures; ensure that the patient entry and outcome data are completed and transmitted to the TSC in a timely manner; ensure the Investigator’s Study File is up to date and complete; be accountable for trial treatments at their site; ensure the trial is conducted in accordance with Good Clinical Practice Guidelines and fulfills all national and local regulatory requirements; allow access to source data for monitoring, audit and inspection; be responsible for archiving all original trial documents, including the data forms, for 5 years after the end of the trial.

**Contacting the Trial Coordinating centre in an emergency**

For urgent enquiries, adverse event reporting and unblinding queries, investigators can contact the 24-h telephone service provided by the TSC. A central telephone number is given in the Investigator’s Study File.

**Sponsorship and trial management**

This research project has no commercial sponsor. The trial will be coordinated by the TSC. The *Sociedad Española de Medicina Intensiva, Crítica y Unidades Coronarias (SEMICYUC)* is funding the run-in costs for this trial and up to 100 patients´recruitment. Full funding is being sought from public and private funding organizations for the main trial. Funding for this trial covers dexamethasone acquisition, pharmacy expenses and central organizational costs only. The design and management of the study are entirely independent of the manufacturers of dexamethasone or the funders.

**Dissemination**

All efforts will be made to ensure that the trial protocol and results arising from the DEXCON TBI trial are published in an established peer-reviewed journal. At least one publication of the main trial results will be made. The success of the trial will be dependent entirely upon the collaboration of the nurses and doctors in the participating hospitals and those who hold key responsibility for the trial. Hence, the credit for the study will be assigned to the key collaborator(s) from each participating site, as it is crucial that those taking credit for the work have actually carried it out. The results of the trial will be reported first to trial collaborators.
